# The evolution of pueriparity maintains multiple paternity in a polymorphic viviparous salamander

**DOI:** 10.1038/s41598-020-71609-3

**Published:** 2020-09-08

**Authors:** Lucía Alarcón-Ríos, Alfredo G. Nicieza, André Lourenço, Guillermo Velo-Antón

**Affiliations:** 1 Departamento de Biología de Organismos y Sistemas, Área de Ecología, Universidad de Oviedo, C/ Valentín Andrés Álvarez S/N, 33071 Oviedo, Spain; 2 Unidad Mixta de Investigación en Biodiversidad (UMIB), CSIC-Universidad de Oviedo-Principado de Asturias, Mieres, Spain; 3 CIBIO/InBIO, Centro de Investigacão em Biodiversidade e Recursos Genéticos, Instituto de Ciências Agrárias de Vairão , Universidade do Porto, R. Padre Armando Quintas 7, 4485-661 Vairão, Portugal; 4 Departamento de Biologia da Faculdade de Ciências da, Universidade do Porto, Rua Campo Alegre, 4169-007 Porto, Portugal

**Keywords:** Animal behaviour, Herpetology, Sexual selection, Genetic variation

## Abstract

The reduction in fecundity associated with the evolution of viviparity may have far-reaching implications for the ecology, demography, and evolution of populations. The evolution of a polygamous behaviour (e.g. polyandry) may counteract some of the effects underlying a lower fecundity, such as the reduction in genetic diversity. Comparing patterns of multiple paternity between reproductive modes allows us to understand how viviparity accounts for the trade-off between offspring quality and quantity. We analysed genetic patterns of paternity and offspring genetic diversity across 42 families from two modes of viviparity in a reproductive polymorphic species, *Salamandra salamandra*. This species shows an ancestral (larviparity: large clutches of free aquatic larvae), and a derived reproductive mode (pueriparity: smaller clutches of larger terrestrial juveniles). Our results confirm the existence of multiple paternity in pueriparous salamanders. Furthermore, we show the evolution of pueriparity maintains, and even increases, the occurrence of multiple paternity and the number of sires compared to larviparity, though we did not find a clear effect on genetic diversity. High incidence of multiple paternity in pueriparous populations might arise as a mechanism to avoid fertilization failures and to ensure reproductive success, and thus has important implications in highly isolated populations with small broods.

## Introduction

The evolution of viviparity entails pronounced changes in individuals’ reproductive biology and behaviour and, by extension, on population dynamics^1–3^. For example, viviparous species often show an increased parental investment compared to oviparous ones because they produce larger and more developed offspring that are protected from external pressures for longer periods within the mother^4–7^. Conversely, viviparity is frequently associated with a reduction in clutch size and fecundity^4,8, 9^. This can affect population effective size (*Ne*) which ultimately may decrease species genetic diversity^10,11^, compromising population viability and adaptive potential to respond to changing environmental pressures^12,13^. Polygamous mating systems, such as polyandry, can minimize the genetic effects resulting from this loss of fecundity. For instance, the ability of females to produce clutches sired by several fathers (i.e. multiple paternity; hereafter MP) may not only increase within-family genetic diversity and *Ne,* but also increase reproductive success through the avoidance of genetic incompatibilities^14–17^.

Amphibians exhibit a great diversity in reproductive strategies^18,19^, and MP has been detected in most life histories^20,21^. However, the reduction of fecundity resulting from the emergence of a novel reproductive mode (e.g. viviparity) might alter the patterns of MP (e.g. its frequency and the number of fathers per clutch) compared to the ancestral reproductive strategy (e.g. oviparity). Although viviparity evolved in each of the three extant orders of amphibians^8^, exploring the relationship between reproductive strategies and patterns of paternity is challenging. This is because reproductive strategies rarely vary at the intra-specific level and comparisons involving phylogenetically distant species may introduce substantial bias and, consequently, prevent robust conclusions. In that sense, the intra-specific polymorphism in reproductive modes exhibited by the fire salamander (*Salamandra salamandra* Linnaeus, 1758) makes it a good system to investigate the potential changes in MP levels and offspring genetic diversity related to the evolution of a novel reproductive strategy.

*Salamandra salamandra* exhibits internal fertilization and two discrete modes of viviparity: (1) an ancestral and more geographically and phylogenetically widespread strategy, larviparity, in which females lay free aquatic larvae^22^; and (2) pueriparity, which is geographically restricted and independently evolved in two subspecies endemic to the Iberian Peninsula (Pleistocene and Holocene origin in *S. s. bernardezi* and *S. s. gallaica*, respectively), and in which females give birth fully metamorphosed juveniles^22–24^. As reported in previous studies, larviparous individuals have on average larger brood sizes (ca. 20–80 aquatic larvae) than pueriparous ones, in which females give birth to ca. 1–35 juveniles, which are generally larger and heavier than aquatic larvae^25–28^. These differences in the offspring are due to several heterochronic processes related to the shift to pueriparity, such as the incomplete fertilization of ovulated eggs, accelerated and asynchronous rates of embryonic development, and active feeding on unfertilized eggs (oophagy) and less developed siblings (adelphophagy)^25^.

Previous studies produced evidence that *S. salamandra* is a polygynandrous species and MP was reported in one larviparous population from Germany of the subspecies *S. s. terrestris*^29,30^. Sperm from multiple mates are accumulated in the spermatheca through a *topping off* mechanism^31^, in which first mates sire the highest proportion of a female’s clutch^30^. Contrary to larviparous salamanders, the presence of MP in pueriparous females is yet to be confirmed. However, one may hypothesize that some of the ontogenetic processes exclusive to pueriparous *S. salamandra* may potentially affect patterns of MP compared to their larviparous counterparts. For example, as shown in other species^32–34^, intrauterine cannibalism occurring exclusively among pueriparous siblings (both oophagy and adelphophagy^25^) reduces brood sizes^26^, but it can also decrease the number of contributing fathers, even if they managed to successfully fertilize some of the female’s eggs.

Here, we used genetic data (microsatellite genotyping) to address two main objectives: 1) to evaluate the presence of MP in the two known independent origins of pueriparity within *S. salamandra* (*S. s. gallaica* and *S. s. bernardezi*); and 2) to compare levels of MP (i.e. incidence of MP and number of sires) between reproductive modes. We hypothesize that pueriparous populations show smaller levels of MP than larviparous ones due to their smaller brood size^26^, the *topping off* mechanism of fertilization^30^, and the presence of intrauterine cannibalism^25^. As a reference of MP for larviparity we used the subspecies *S. s. terrestris,* which occurs throughout central Europe, and is phylogenetically close to *S. s. gallaica*^35^*.* As secondary objectives, we also 3) compared patterns of MP between pueriparous *S. s. bernardezi* and *S. s. gallaica,* hypothesizing a higher incidence of MP and number of sires in the latter, as a result of their larger body size and a more recent evolution of pueriparity, and 4) evaluated whether a higher number of siring males increased the fecundity of females and the genetic diversity of their offspring^14,36^. Finally, and as pilot study, we compared patterns of MP between natural births and dissections at early developmental stages in pueriparous populations, expecting a lower number of sires per clutch in natural births due to the occurrence of intrauterine cannibalism at later stages of pregnancy.

## Material and methods

**Figure 1:**
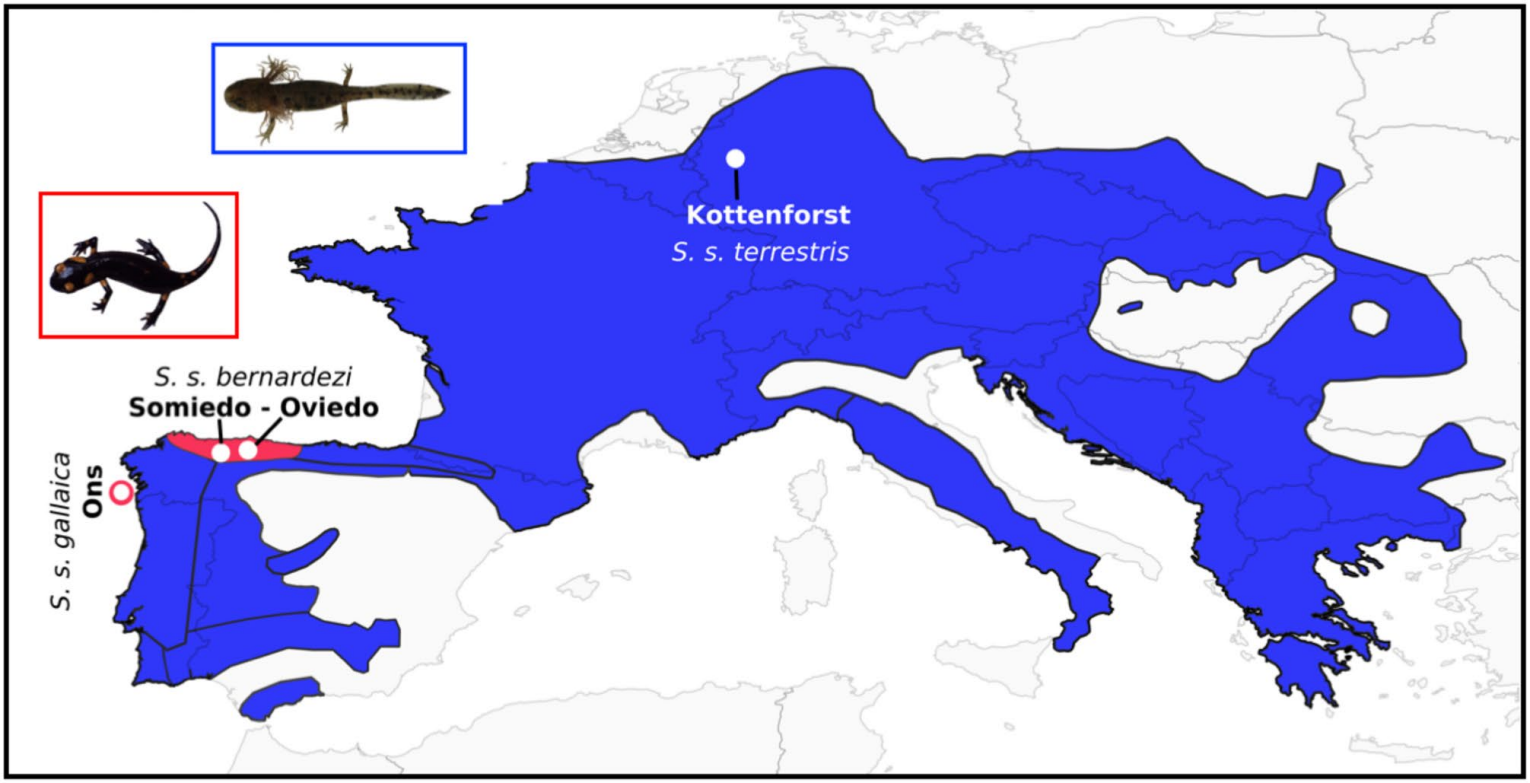
Map displaying the range of distribution of *S. salamandra* and the location of the studied populations. The range of distribution of *S. salamandra* (in blue and red), includes the distribution of all larviparous subspecies (blue area) and both pueriparous nuclei (red). Black lines delimit the area of distribution of different subspecies described within *Salamandra salamandra*. Pictures are not scaled.

We sampled 18 pueriparous females from two *S. s. bernardezi* populations (Oviedo, N = 5; Somiedo, N = 5) and one of the two extant pueriparous *S. s. gallaica* populations (Ons Island; N = 8) (Fig. 1; Table 1 and Supplementary Table S1). During the course of the study we could not observe and collect any gravid females from the rare and small insular pueriparous population of *S. s. gallaica* in San Martiño^37^. Between 2015 and 2017 we collected six and four gravid females of *S. s. bernardezi* (three from each population) and *S. s gallaica,* respectively, and transported them to laboratory facilities at the University of Oviedo (Spain; in the case of *S. s. bernardezi*) and to the Research Centre in Biodiversity and Genetic Resources (CIBIO-InBIO, Porto, Portugal; in the case of *S. s. gallaica*). Individuals were placed in individual terraria (60 × 30 × 40 cm; L × W × H) containing coconut fibre as substrate, a container with water, moss, and shelters (bricks or barks). We fed them twice a week with crickets (*Acheta* sp.) or flour worms (*Tenebrio* sp.). After parturition, both females and their offspring were released at their place of capture.

**Table 1:** Summary results for each studied population.

Subspecies	Population	N_F_	N_off_	N_fathers_	Range	Incid (%)	%Sired	H_o_	N_A_	A_R_	R_off_
*S. s. bernardezi*	Oviedo	5	11.2 ± 1.64	1.8 ± 0.84	1–3	60	86.8 ± 14.55	0.67 ± 0.07	2.80 ± 0.31	1.88 ± 0.09	0.65 ± 0.08
	Somiedo	5	6.60 ± 3.29	1.6 ± 0.55	1–2	60	86.4 ± 14.91	0.74 ± 0.09	3.04 ± 0.19	2.05 ± 0.15	0.54 ± 0.11
*S. s. gallaica*	Ons	8	18.5 ± 8.33	3.8 ± 2.05	1–7	88	63.8 ± 22.24	0.55 ± 0.08	2.84 ± 0.53	1.96 ± 0.62	0.59 ± 0.07
*S. s. terrestris*	Kottenforst	24	24.5 ± 13.02	2.0 ± 1.18	1–5	54	86.7 ± 16.43	0.63 ± 0.09	2.70 ± 0.48	2.12 ± 1.53	0.46 ± 0.13

Results on multiple paternity (mean ± s.d.): number of females (N_F_), total clutch size (N_off_), number of fathers per clutch (N_fathers_), range of the number of fathers (Range), proportion of multiple-sired families (Incid.), proportion sired by the most successful male (%Sired). Mean genetic diversity parameters within each population (± s.e.m.): observed heterozygosity (H_o_); mean number of alleles (N_A_); unbiased allelic richness (A_R_); mean pairwise relatedness within each clutch (R_off_).

To explore whether the number of siring males varied across gestation stages, we sampled eight more females (two from each *S. s. bernardezi* population and four from Ons) at the earliest gestation stage possible and sacrificed them by an overdose of anaesthesia (benzocaine; Ethyl 4-aminobenzoate; Sigma-Aldrich, Darmstadt, Germany. Product number: E1501. Ref.: 112909). After dissections, we recorded the uterus (i.e. right or left), clutch size, and the stage of development of each offspring (i.e. embryo, larvae, juvenile). Each individual was stored in pure ethanol for DNA analysis. Salamanders were captured and processed under collection permits provided by regional or national governments (Galicia, Ref. 410/2015 and EB016/2018; Asturias, NºEXPTE: 2017/001208; 2018/007781; 2018/2115), and the study was approved by the ethics committee Research Ethics Committee of the University of Oviedo (PROAE 10/2017). All applicable national and institutional guidelines for the care and use of animals were followed.

We used microsatellite markers to infer patterns of MP (i.e. number of sires and incidence of MP), and characterize within-family genetic diversity. Tissue samples for DNA analysis were collected from a toe-clip in the case of females, and a tail-clip from juveniles. We genotyped 11 microsatellites^38,39^, following the conditions described in^40,41^ (see Supplementary Information and Supplementary Table S2 for details). For the larviparous reproductive mode, we used a comprehensive dataset obtained for *S. s. terrestris* and published by ref.^30,42^. This dataset includes a total of 591 larvae and 24 females genotyped for a higher number of microsatellite markers. For comparison purposes, we filtered these genotypic profiles to the same 11 loci employed in our study for pueriparous individuals. Although we are aware the optimal design should have included data from nearby larviparous populations of *S. s. gallaica,* we argue that our comparisons are valid because *S. s. gallaica* and *S. s. terrestris* are phylogenetically closely related^35^. Indeed, phylogenomic analyses show *S. s. terrestris* as a recent expanded population from *S. s. gallaica* with very shallow levels of genetic divergence (unpublished genomic data and ref.^35^).

To infer the number of siring males in each family we estimated the most likely number of fathers in COLONY 2.0.6.4.^43^, a software which implements a maximum likelihood method to infer parentage based on individual multilocus genotypes. We set the species as dioecious and diploid, assumed polygamy for both sexes, no inbreeding, the maternal genotype and maternal sibship was considered known a priori, and no candidate father genotype was included. We applied the maximum likelihood approach with high likelihood precision and two very long runs with different random number seed in each population separately. We provided neither known population allele frequency nor sibship size prior. As we were very conservative during allele scoring and re-amplified a number of samples to check for possible errors (see Supplementary Information), we assumed a minimum error rate of 0.0001. To characterize the genetic diversity of each brood, we calculated the observed heterozygosity (H_o_), mean number of alleles (N_A_), and mean relatedness of offspring per female (R_offsspring_) in GenAlex v 6.503^44^. As N_A_ could be highly affected by differences in brood size, we calculated the unbiased allelic richness (A_R_) using a rarefaction method implemented in HP-RARE^45^.

We compared the mean clutch size (N_offspring_) and the mean number of fathers (N_fathers_) among the studied subspecies, modes of reproduction, and stage of gestation (i.e. natural births vs. dissections) using permutation tests with 1,000 resamplings without replacement (α = 0.05). Additionally, for each subspecies, we used non-parametric Kendall correlations to test the association between MP (N_fathers_) and variables related to female’s fecundity (N_offspring_), and offspring genetic diversity (H_o_, N_A_, A_R_, R_offspring_). Because the mean number of alleles (N_A_) is often highly affected by N_offspring_, we also tested for the correlation between both variables to enable us to examine whether a putative high correlation between N_fathers_ and N_A_ is an artefact of the varying N_offspring_ observed among the studied females. All statistical analyses were performed in R software^46^.

## Results

We obtained 237 offspring from 18 pueriparous females. Four individuals from the original larviparous dataset were excluded due to incongruences with the mother (H04_BT_373) or high presence of missing data (H05_BT_565, H06_BT_566, H07_BT_567), resulting in a final dataset of 587 offspring from the 24 larviparous females. All other individuals were unambiguously assigned to their mothers.

**Figure 2:**
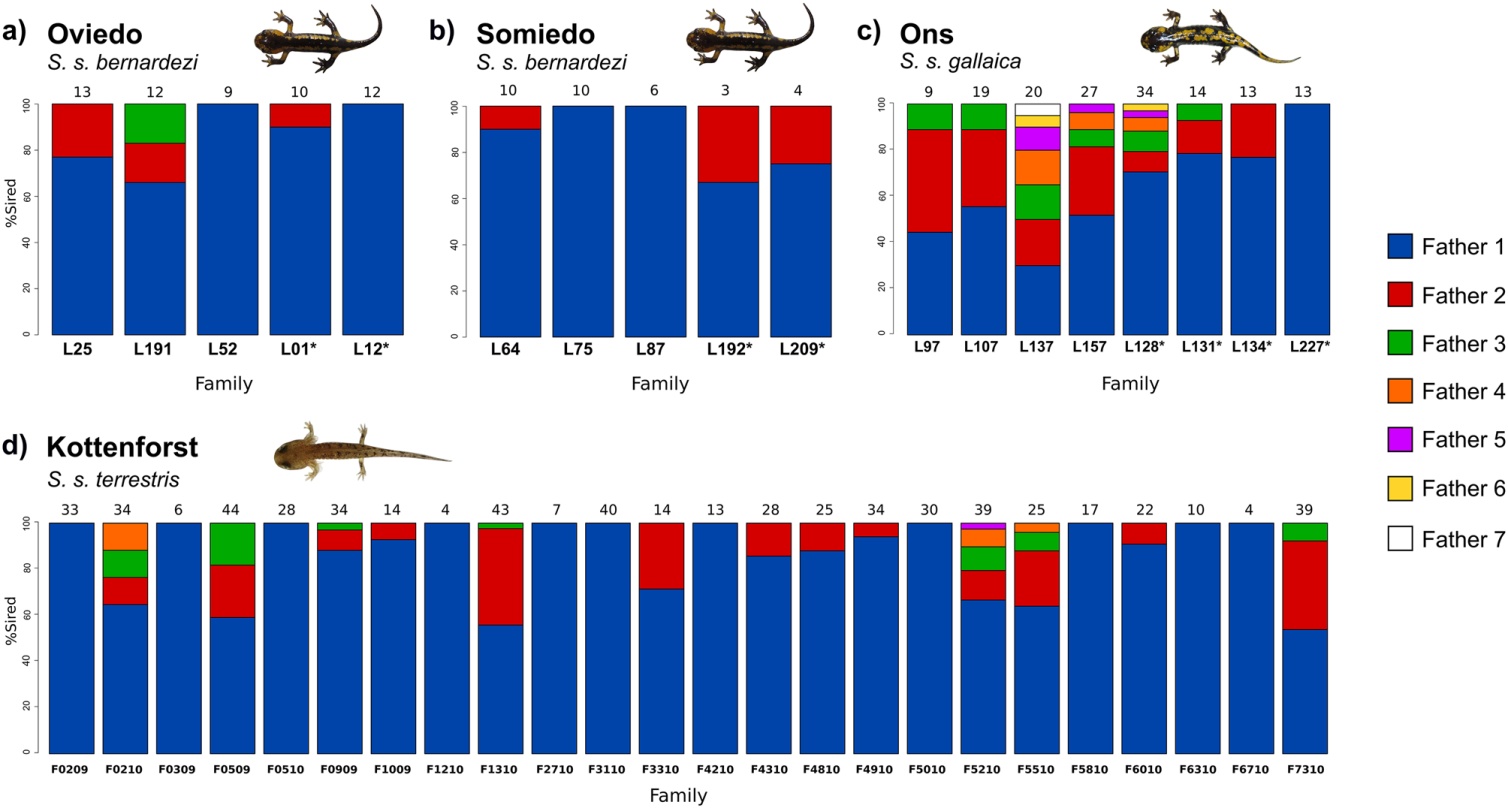
Number of fathers per clutch and percentage of the offspring sired by each father. The top row of barplots illustrate the three studied pueriparous populations, while the bottom barplot represents the larviparous population. Above each bar, the number of offspring per clutch is displayed. Asterisks (*) denote families that came from dissections. Kottenforst original data from ref.^30^.

We found evidence for MP in the three study subspecies (*S. s. bernardezi*, *S. s. gallaica*, *S. s. terrestris*), with incidence of MP varying between 54 and 88% (highest in pueriparous *S. s. gallaica*) and N_fathers_ ranging between 1 and 7 in pueriparous populations, and 1 and 5 in the larviparous population (Table 1; Fig. 2). In cases where N_fathers_ > 1, a single male sired on average 71.4% (± 16.4%) of the offspring. The *S. s. bernardezi* population of Oviedo and the *S. s. gallaica* population of Ons exhibited the lowest genetic diversity and the highest relatedness among the studied populations (Table 1).

Because they belong to the same subspecies and have reduced sample size, females from both pueriparous *S. s. bernardezi* populations were pooled (N_offspring_ = 8.9, N_fathers_ = 1.7) and compared with both pueriparous *S. s. gallaica* and larviparous *S. s. terrestris*. Permutation tests revealed *S. s. gallaica* (N_offspring_ = 18.5, N_fathers_ = 3.8) exhibited a significantly higher N_offspring_ (*P* = 0.001) and N_fathers_ (*P* = 0.001) than *S. s. bernardezi*. Comparisons involving different reproductive modes showed that *S. s. terrestris* (N_offspring_ = 24.5, N_fathers_ = 2.0) has a significantly higher N_offspring_ than *S. s. bernardezi* (*P* = 0.001), but not than *S. s. gallaica* (*P* = 0.869). The N_fathers_ was similar between *S. s. terrestris* and *S. s. bernardezi* (*P* = 0.187), while the offspring of *S. s. gallaica* females appeared to be sired by a higher number of fathers than their larviparous counterparts (*P* = 0.005) (Table 1; Supplementary Table S3).

All pairwise comparisons involving pueriparous subspecies include data from natural births and dissections because permutation tests did not show differences (*P* > 0.05) in N_offspring_ or N_fathers_ between both sampling methods (Table 1; Supplementary Table S4). The inclusion of females sampled at different stages of pregnancy could affect comparisons involving pueriparous subspecies and the larviparous *S. s. terrestris*, because the data for the latter were collected only from natural births. However, when excluding dissections, the results from permutation tests do not differ from those including all sampled pueriparous females (Supplementary Table S5).

We did not find a significant relationship between N_A_ and N_offspring_ among the study subspecies (*S. s. bernardezi: τ* = − 0.02, *P* = 0.93; *S. s. gallaica: τ* = 0.33, *P* = 0.26; *S. s. terrestris: τ* = 0.18, *P* = 0.23), therefore, we also tested the association between N_fathers_ and N_A_. Correlation tests revealed a significant positive relationship between N_fathers_ and female’s fecundity in the larviparous population and the pueriparous *S. s. gallaica*, but not in pueriparous *S. s. bernardezi* (Table 2). While heterozygosity in *S. s. bernardezi* seemed negatively correlated with multiple paternity, N_A_ varied positively with N_fathers_ in *S. s. gallaica* and *S. s. terrestris*. Indeed, in the latter subspecies, relatedness appears to decrease as the number of fathers increases (Table 2).

**Table 2:** Results of the Kendall correlation tests. Relationship (Kendall’s tau, *τ*) between the number of sires (N_fathers_) over clutch size (N_offspring_) and offspring genetic diversity values (H_o_, N_A_, A_R_, R_offspring_).

	N_offspring_	H_o_	N_A_	A_R_	R_offspring_
***S. s. bernardezi***					
*τ*	0.11	**− 0.59**	0.06	− 0.47	0.28
*P*	0.684	**0.035**	0.839	0.158	0.315
***S. s. gallaica***					
*τ*	**0.62**	0.04	**0.72**	0.12	− 0.39
*P*	**0.040**	0.898	**0.016**	0.694	0.199
***S. s. terrestris***					
*τ*	**0.48**	0.07	**0.59**	0.19	**− 0.38**
*P*	**0.003**	0.671	** < 0.001**	0.222	**0.018**

Significant p-values (*P*; α = 0.05) and Kendall’s tau are in bold.

## Discussion

This study confirmed the occurrence of MP in pueriparous salamanders, a reproductive strategy restricted to only two urodele genera: *Salamandra* and *Lyciasalamandra*^47–49^. While previous studies documented a polygamous mating system in the pueriparous *S. atra*^50–52^, to the best of our knowledge, the occurrence of MP in any pueriparous species had never been proven.

Contrary to our expectations, the shift to pueriparity seems to maintain, and even increase, the incidence of MP and the number of sires per clutch (Table 1; Fig. 2) despite the smaller brood sizes compared to larviparous salamanders. This result is in agreement with previous studies showing no association between MP and clutch size because similar or even lower levels of MP are known among species of amphibians producing large clutches (e.g. refs.^31,53–55 ^)*.* Moreover, benefits of polyandry may vary among reproductive modes^56^. In less fecund reproductive modes, female investment per offspring is generally high, and reproductive failures due to genetic incompatibilities imply higher costs compared to more fecund strategies^56,57^. Within *S. salamandra,* larviparous progeny rely solely on their yolk provisions, but in pueriparous populations, fecundity is reduced in favour of a lower number of more developed descendants because nutrient provisioning occurs through the availability of both arrested eggs and less developed siblings^25,27^. Accordingly, reproductive costs due to genetic incompatibilities or mating with sterile males are likely higher in pueriparous females and, therefore, multiple mating (and MP) may be under selection to reduce these costs^58^. Furthermore, in accordance to previous studies we found a similar pattern of reproductive skew towards a dominant male in polyandrous broods in both reproductive modes, probably resulting from a *topping-off* mechanism of sperm storage^31^, in which the first mate fertilizes the majority of eggs^30^. Although we found no significant differences in clutch size between the larviparous *S. s. terrestris* and the pueriparous insular *S. s. gallaica*, the larviparous mainland *S. s. gallaica* generally have larger clutches^26^.

Although similar heterochronic processes are thought to mediate pueriparity in both *S. s. bernardezi* and *S. s. gallaica*^26^, clutch size, number of fathers, and incidence of MP are higher in the latter (Table 1). The differences observed between the studied pueriparous subspecies might result from the more recent transition to pueriparity in *S. s. gallaica* (ca. 8,000 years ago^59^), which may have led to the retention of some ‘larviparous’ traits associated with reproduction. Additionally, the population-specific traits of this insular population and differences in evolutionary history (e.g. founder effects, isolated, low genetic diversity)^37,59,60^ may have also contributed to the differences in patterns of paternity observed between insular and continental pueriparous populations. Indeed, the particularly low levels of genetic diversity and higher levels of inbreeding observed in Ons^24,60^ may increase the risk of reproductive failures and, therefore, MP in Ons may be under selection to increase reproductive success^16^. Moreover, we found in this population the highest number of males (7) siring a single clutch in *S. salamandra*, which is remarkable even at the wider taxonomic spectrum of internally fertilizing vertebrates^57,61,62^. Nonetheless, further research is needed to understand whether MP boosts reproductive success in the fire salamander, and its relationship with other population traits, such as inbreeding.

Salamander males do not provide parental care or extra resources to females or their offspring. However, females may obtain genetic benefits from polyandry. Multiple paternity has been suggested as a mechanism of genetic compensation that helps maintaining high *Ne/N* ratios and relatively high levels of genetic diversity^16,63,64^, such as for example, in a small pueriparous population of *S. s. bernardezi*^41,65^, and in an overexploited population of rockfish^66^. Our results appear to corroborate the aforementioned premises; a higher number of fathers appears to be associated not only with an increase in allele diversity within *S. s. gallaica* and in the larviparous population, but also with a reduction in the offspring genetic relatedness in *S. s. terrestris*. This potentially suggests MP boosts genetic diversity, though the negative relationship observed between H_o_ and N_fathers_ in *S. s. bernardezi,* together with the reduced sample size, call for caution when interpreting these results.

Another potential benefit of polyandry is an increase in clutch size^67^. We detected a positive relationship between the number of sires and the number of offspring in the larviparous population (see also ref.^30^). This pattern holds in the pueriparous *S. s. gallaica*, but not in *S. s. bernardezi*, suggesting that small sample size and other factors not considered in this study can be also influencing fecundity, such as the body size of females and offspring^8^, thus preventing us from making robust predictions about the relationship between polyandry and clutch size. In addition, it is worth considering that in pueriparous systems, the high production of aborted eggs and cannibalistic events reduce brood size^68^, and thus, this variable likely misrepresents the amount of successful fertilizations.

Finally, we did not detect substantial differences in clutch sizes or the number of sires between natural births and dissections, although it should be acknowledged that most dissections were performed at a relatively advanced stage of pregnancy. Thus, sampling more females at earlier stages of pregnancy, where most arrested eggs were not yet cannibalized, will be crucial to clarify how ontogenetic processes associated with pueriparity affect the incidence and frequency of MP. For instance, in the polygamous pueriparous *S. atra* species, discordances between the number of mating males and sires are expected because only one or two completely developed offspring are delivered after a long period of gestation over which the main source of nourishment consists on aborted eggs^50,52,69^.

The present study constitutes the first step towards the understanding of the potential relationship between MP and alternative life histories in *S. salamandra.* Specifically, we showed pueriparous populations of *S. salamandra* exhibit identical or higher rates of multipaternity, even though their fecundity is lower. Nevertheless, we cannot rule out that some other factors may have also influenced our results. The lack of replicates for both larviparous populations and the pueriparous *S. s. gallaica* limit our ability in disentangling the effects of population-specific traits and reproductive mode in the observed patterns of multipaternity. Specifically, our data do not allow us to evaluate accurately whether other ecological, geographical and demographic traits influence MP^70–72^. Indeed, despite the potential benefits that MP entails in an isolated and inbred population, the high rates of MP observed in the insular population of Ons can reflect a relaxation of the potential constraints determining the acquisition of mates (e.g. population density, rates of individuals encounters, territoriality)^37,62,73,74^. Hence, future studies should extend sampling to cover as much diversity within the species as possible and consider the potential effects of geographic and populational variability on MP.

Overall, this study constitutes a relevant contribution to the understanding on how reproductive strategies evolve to account for the trade-off between offspring quality and quantity. Although other factors might be involved, our results appear to suggest the evolution of a derived reproduction mode may affect other traits to compensate the loss of fecundity and ensure reproductive success.

## Supplementary information

Supplementary information

## Data Availability

Datasets used in the present study can be found in Figshare (10.6084/m9.figshare.12014580).
