# Supplementary material for: The evolution of pueriparity maintains multiple paternity in a polymorphic viviparous salamander
Source: Sci Rep. 2020 Sep 8;10:14744. doi: 10.1038/s41598-020-71609-3 (PMC7479106; doi:10.1038/s41598-020-71609-3)
Supplement: Supplementary file 1 — Supplementary information [file 41598_2020_71609_MOESM1_ESM.pdf]

Supplementary Material:

The evolution of pueriparity maintains multiple paternity in a polymorphic viviparous salamander

Lucía Alarcón-Ríos[1*], Alfredo G. Nicieza[1,2], André Lourenço[3,4], Guillermo Velo-Antón[3*]

Affiliations:
1 Departamento de Biología de Organismos y Sistemas, Área de Ecología, Universidad de Oviedo, Oviedo, Spain.
2 Unidad Mixta de Investigación en Biodiversidad (UMIB), CSIC-Universidad de Oviedo-Principado de Asturias, Mieres, Spain
3 CIBIO/InBIO, Centro de Investigacão em Biodiversidade e Recursos Genéticos, Instituto de Ciências Agrárias de Vairão 7, Universidade do Porto, Vairão, Portugal
4 Departamento de Biologia da Faculdade de Ciências da Universidade do Porto. Rua Campo Alegre, 4169-007 Porto, Portugal

*Corresponding authors

Corresponding authors:

Alarcón-Ríos L
E-mail: alarconrioslucia@gmail.com
ORCID: https://orcid.org/0000-0003-4015-9609
Dpto. Biología de Organismos y Sistemas, Área de Ecología.
Universidad de Oviedo
C/ Valentín Andrés Álvarez s/n,
33071, Oviedo (Principado de Asturias). España

Velo-Antón G
E-mail: guillermo.velo@gmail.com
ORCID: https://orcid.org/0000-0002-9483-5695
CIBIO-InBIO, Centro de Investigação em Biodiversidade e Recursos Genéticos da Universidade do Porto
Instituto de Ciências Agrárias de Vairão
R. Padre Armando Quintas 7
4485-661, Vairão. Portugal

Table S1. Details of each studied family: subspecies and population of origin of each female, sample type (B: natural birth; D: dissection), number of juveniles (N_J), larvae (N_L), embryos (N_E), and clutch size (N_{off}). Results regarding multiple paternity: number of fathers in each clutch ($N_{fathers}$); the table also shows the proportion of offspring sired by the most successful father (%Sired), with the proportion of the second most successful father being also displayed in parentheses when percentages are similar. Analyses of genetic diversity within each family: observed heterozygosity (H_o); mean number of alleles (N_A); unbiased allelic richness (A_R); mean pairwise relatedness within each clutch (R_{off}).

Subspecies	Pop.	Female	Sample	N_J	N_L	N_E	N_{off}	$N_{fathers}$	%Sired	H_o	N_A	A_R	R_{off}
S. s. bernardezi	Oviedo	L25	B	13	0	0	13	2	77	0.72	3.18	1.91	0.64
S. s. bernardezi	Oviedo	L191	B	11	1	0	12	3	67	0.50	2.91	1.81	0.64
S. s. bernardezi	Oviedo	L52	B	9	0	0	9	1	100	0.77	2.91	2.02	0.55
S. s. bernardezi	Oviedo	L01	D	0	5	5	10	2	90	0.68	2.36	1.81	0.76
S. s. bernardezi	Oviedo	L12	D	0	12	0	12	1	100	0.69	2.64	1.86	0.68
S. s. bernardezi	Somiedo	L64	B	5	5	0	10	2	90	0.67	3.00	1.90	0.60
S. s. bernardezi	Somiedo	L75	B	6	4	0	10	1	100	0.85	3.18	2.13	0.54
S. s. bernardezi	Somiedo	L87	B	6	0	0	6	1	100	0.77	2.91	2.02	0.58
S. s. bernardezi	Somiedo	L192	D	3	0	0	3	2	67	0.80	3.27	2.26	0.35
S. s. bernardezi	Somiedo	L209	D	3	1	0	4	2	75	0.61	2.82	1.92	0.62
S. s. gallaica	Ons	L97	B	9	0	0	9	3	45 (45)	0.60	2.73	3.48	0.56
S. s. gallaica	Ons	L107	B	18	0	0	18	3	56	0.56	2.46	1.7	0.65
S. s. gallaica	Ons	L137	B	19	1	0	20	7	30 (20)	0.67	3.91	1.94	0.43
S. s. gallaica	Ons	L157	B	27	0	0	27	5	52	0.43	2.64	1.62	0.64
S. s. gallaica	Ons	L128	B+D	26	8	0	34	6	71	0.54	3.27	1.71	0.59
S. s. gallaica	Ons	L131	B+D	13	1	0	14	3	79	0.49	2.91	1.7	0.62
S. s. gallaica	Ons	L134	D	12	1	0	13	2	77	0.62	2.55	1.79	0.61
S. s. gallaica	Ons	L227	D	7	6	0	13	1	100	0.52	2.27	1.7	0.64
S. s. terrestris	Kottenforst	F0209	B	0	33	0	33	1	100	0.458	2.09	1.62	0.59
S. s. terrestris	Kottenforst	F0210	B	0	34	0	34	4	65	0.565	3.00	1.76	0.43
S. s. terrestris	Kottenforst	F0309	B	0	6	0	6	1	100	0.636	2.27	1.77	0.55
S. s. terrestris	Kottenforst	F0509	B	0	44	0	44	3	59	0.669	3.73	1.97	0.25
S. s. terrestris	Kottenforst	F0510	B	0	28	0	28	1	100	0.598	2.46	1.77	0.46
S. s. terrestris	Kottenforst	F0909	B	0	34	0	34	3	88	0.811	3.27	1.93	0.42
S. s. terrestris	Kottenforst	F1009	B	0	14	0	14	2	93	0.633	2.46	1.79	0.54
S. s. terrestris	Kottenforst	F1210	B	0	4	0	4	1	100	0.841	3.00	2.13	0.11
S. s. terrestris	Kottenforst	F1310	B	0	43	0	43	3	56 (42)	0.533	2.91	1.75	0.44
S. s. terrestris	Kottenforst	F2710	B	0	7	0	7	1	100	0.571	2.46	1.75	0.53
S. s. terrestris	Kottenforst	F3110	B	0	40	0	40	1	100	0.552	2.00	9.28	0.67
S. s. terrestris	Kottenforst	F3310	B	0	14	0	14	2	71	0.670	3.00	1.83	0.43
S. s. terrestris	Kottenforst	F4210	B	0	13	0	13	1	100	0.555	2.36	1.74	0.49
S. s. terrestris	Kottenforst	F4310	B	0	28	0	28	2	86	0.679	2.82	1.85	0.39
S. s. terrestris	Kottenforst	F4810	B	0	25	0	25	2	88	0.625	2.64	1.72	0.58
S. s. terrestris	Kottenforst	F4910	B	0	34	0	34	2	94	0.464	2.27	1.6	0.62
S. s. terrestris	Kottenforst	F5010	B	0	30	0	30	1	100	0.563	2.18	1.64	0.62
S. s. terrestris	Kottenforst	F5210	B	0	39	0	39	5	67	0.552	3.18	1.86	0.32
S. s. terrestris	Kottenforst	F5510	B	0	25	0	25	4	64	0.715	3.27	1.92	0.34
S. s. terrestris	Kottenforst	F5810	B	0	17	0	17	1	100	0.717	2.73	1.92	0.40
S. s. terrestris	Kottenforst	F6010	B	0	22	0	22	2	91	0.777	3.64	2.03	0.24
S. s. terrestris	Kottenforst	F6310	B	0	10	0	10	1	100	0.605	2.27	1.71	0.56
S. s. terrestris	Kottenforst	F6710	B	0	4	0	4	1	100	0.636	2.18	1.75	0.58
S. s. terrestris	Kottenforst	F7310	B	0	39	0	39	3	58 (38)	0.608	2.73	1.8	0.47

Laboratory procedures

A total of 11 microsatellites (Sal29, SalE12, SalE7, SalE5, SalE2, SalE06, Sal3, SalE08 [1]; SST-B11, SST-C3, SST-G9 [2]), distributed in three optimized multiplexes (panels S2, S3, S4) (Table S2), were amplified through polymerase chain reaction (PCR) following the conditions described in [3]. Each multiplex mix contained distilled H_2O, fluorescently labelled forward (6-FAM, VIC, NED or PET; Table S2 for sequence details) and reverse primers. Each PCR reaction contained a total volume of 10–11 µl: 5 µl of Multiplex PCR Kit Master Mix (QIAGEN), 3 µl of distilled H_2O, 1 µl of primer multiplex mix and 1–2 µl of DNA extract (~ 50 ng/µl). To identify possible contaminations, a negative control was employed. PCR touchdown cycling conditions were equal in all multiplexes reactions: the reaction started with an initial step at 95 °C for 15 min, 19 cycles at 95 °C for 30 s, 90 s of annealing at 65 °C (decreasing 0.5 °C each cycle), 72 °C for 40 s, followed by 25 cycles of 95 °C for 30 s, 56 °C for 60 s, 72 °C for 40 s, and ended with a final extension of 30 min at 60 °C. To determine the relative size of fragments, the DNA Size Standard LIZ 500 DSMO-100 (MCLAB) was employed. Quality PCR products were verified on a 2% agarose gel run on an ABI3130XL capillary sequencer (Applied Biosystems). Alleles were scored in GENEMAPPER 4.0 (Applied Biosystems). To reduce the potential influence of allele dropout and false alleles, we scored only alleles exhibiting clear fluorescence peaks higher than 100 relative fluorescent units. To increase the likelihood of amplification, we amplified in duplex reactions those females (mothers) samples in which any microsatellite marker failed to amplify or exhibited dubious allelic profiles (e.g. peak artefacts). Cycling conditions are the same as those described for multiplexes. Then, we manually checked for correspondences between females' and their offspring's genotype. If any incongruence was detected (e.g. any descendant did not present any allele form the mother at any loci), we reamplified females' loci in uniplex. If incongruences persisted, we reamplified incongruent loci of the offspring in duplexes or uniplexes. Finally, if reamplified loci (from both the female and offspring) were still incongruent, we recorded those offspring genotypes as missing data.

Table S2. Details of the 11 microsatellites [1,2] used in this study and information on multiplex arrangement. Original published primers forward and reverse sequences, fluorescently labelled oligonucleotides used as template for modified forward primers and the concentration of primer forward and reverse used on 10 µl PCR reactions are displayed. This table is adapted from Supplementary Material 2 of ref. [4] and Appendix 2 in ref. [3].

**Locus**	**Multiplex**	**Label***	**Primer forward (5' – 3')**	**Primer reverse (5' – 3')**	**PF concentration PCR (µM)**	**PR concentration PCR (µM)**
Sal29 [1]	Panel S2	6-FAM	CTCTTTGACTGAACCAGAACCCC	GCCTGTCGGCTCTGTGTAACC	0.08	0.8
SST-B11 [2]	Panel S2	PET	TCAAACGGTGCCAAAGTTATTAG	TTAATTGGCAGTTTTCTTTCCAG	0.02	0.2
SalE12 [1]	Panel S2	VIC	CTCAGGAACAGTGTGCCCCAAATAC	CTCATAATTTAGTCTACCCTCCCAC	0.008	0.08
SST-C3 [2]	Panel S3	PET	CCGTTTGAGTCACTTCTTTCTTG	TTGCTTTACCAACCAGTTATTGTC	0.014	0.14
SalE7 [1]	Panel S3	NED	TTTCAGCACCAAGATACCTCTTTTG	CTCCCTCCATATCAAGGTCACAGAC	0.008	0.08
SalE5 [1]	Panel S3	6-FAM	CCACATGATGCCTACGTATGTTGTG	CTCCTGTTTACGCTTCACCTGCTCC	0.006	0.06
SalE2 [1]	Panel S3	VIC	CACGACAAAATACAGAGAGTGGATA	ATATTTGAAATTGCCCATTTGGTA	0.03	0.3
SalE06 [1]	Panel S4	VIC	GGACTCATGGTCACCCAGAGGTTCT	ATGGATTGTGTCGAAATAAGGTATC	0.012	0.12
Sal3 [1]	Panel S4	6-FAM	CTCAGACAAGAAATCCTGCTTCTTC	ATAAATCTGTCCTGTTCCTAATCAG	0.012	0.12
SalE8 [1]	Panel S4	NED	GCAAAGTCCATGCTTTCCCTTTCTC	GACATACCAAAGACTCCAGAATGGG	0.008	0.08
SST-G9 [2]	Panel S4	NED	CCTCGTCAGGGGTTGTAGG	CTTTCCAGGAAGAAACTGAGATG	0.008	0.08

*An extra number of base pairs were added at the 5' end of the original sequence of forward primers in order to allow binding of four different fluorescent labelled oligonucleotides (6-FAM - TGT AAA ACG ACG GCC AGT; VIC - TAA TAC GAC TCA CTA TAG GG; NED - TTT CCC AGT CAC GAC GTT G; PET - GAT AAC AAT TTC ACA CAG G)

Table S3. Results of the permutation tests for the difference in the means of $N_{offspring}$ and $N_{fathers}$ among the considered subspecies. Each permutation test evaluated the alternative hypothesis the mean in $N_{offspring}$ and $N_{fathers}$ for a given subspecies was higher (>) than the other subspecies being compared. The values in columns $N_{offspring}$ and $N_{fathers}$ represent the p-values (α=0.05). Significant p-values ($P < 0.05$) are in bold.

Alternative hypothesis	$N_{offspring}$	$N_{fathers}$
S. s. gallaica > *S. s. bernardezi*	**0.001**	**0.001**
S. s. terrestris > *S. s. bernardezi*	**0.001**	0.187
S. s. gallaica > *S. s. terrestris*	0.869	**0.005**

Table S4. Results of the permutation tests for the difference in the means of $N_{offspring}$ and $N_{fathers}$ between dissections and natural births for *S. s. bernardezi* and *S. s. gallaica*. Each permutation test evaluated the alternative hypothesis that the mean in $N_{offspring}$ and $N_{fathers}$ for dissections was higher (>) than in natural births for each pueriparous subspecies (*S. s. bernardezi* and *S. s. gallaica*). The values in columns $N_{offspring}$ and $N_{fathers}$ represent the p-values (α=0.05). No p-value was statistically significant.

Alternative hypothesis	$N_{offspring}$	$N_{fathers}$
S. s. bernardezi (dissections) > *S. s. bernardezi* (births)	0.839	0.271
S. s. gallaica (dissections) > *S. s. gallaica* (births)	0.487	0.802

Table S5. Results of the permutation tests for the difference in the means of $N_{offspring}$ and $N_{fathers}$ among the considered subspecies excluding data from dissections. Each permutation test evaluated the alternative hypothesis that the mean in $N_{offspring}$ and $N_{fathers}$ for a given subspecies was higher (>) than the other subspecies being compared. The values in columns $N_{offspring}$ and $N_{fathers}$ represent the p-values (α=0.05). Significant p-values ($P < 0.05$) are in bold.

Alternative hypothesis	$N_{offspring}$	$N_{fathers}$
S. s. gallaica > *S. s. bernardezi*	**0.016**	**0.001**
S. s. terrestris > *S. s. bernardezi*	**0.005**	0.196
S. s. gallaica > *S. s. terrestris*	0.792	**0.002**

References

1. Steinfartz, S., Kuesters, D. & Tautz, D. Isolation and characterization of polymorphic tetranucleotide microsatellite loci in the fire salamander *Salamandra salamandra* (Amphibia: Caudata). *Mol. Ecol. Notes* **4**, 626–628 (2004).
2. Hendrix, R., Susanne Hauswaldt, J., Veith, M. & Steinfartz, S. Strong correlation between cross-amplification success and genetic distance across all members of ‘True Salamanders’ (Amphibia: Salamandridae) revealed by *Salamandra salamandra*-specific microsatellite loci. *Mol. Ecol. Resour.* **10**, 1038–1047 (2010).
3. Lourenço, A., Antunes, B., Wang, I. J. & Velo-Antón, G. Fine-scale genetic structure in a salamander with two reproductive modes: Does reproductive mode affect dispersal? *Evol. Ecol.* **32**, 699–732 (2018).
4. Álvarez, D., Lourenço, A., Oro, D. & Velo-Antón, G. Assessment of census (N) and effective population size (*Ne*) reveals consistency of *Ne* single-sample estimators and a high *Ne*/N ratio in an urban and isolated population of fire salamanders. *Conserv. Genet. Resour.* **7**, 705–712 (2015).
